# Disseminated nodulo-ulcerative lesions associated with chronic liver disease

**DOI:** 10.1016/j.jdcr.2023.08.006

**Published:** 2023-08-19

**Authors:** Francilene Moreira Peçanha, Katia Santana Cruz, Flávio Luis Dantas Portela, Vírginia Vilasboas, Andréa de Souza Cavalcante

**Affiliations:** a Dermatology Resident – PGY3, Tropical Medicine Foundation – Dr Heitor Vieira Dourado, Manaus, Amazonas, Brazil; b Pharmacist, Tropical Medicine Foundation – Dr Heitor Vieira Dourado, Manaus, Amazonas, Brazil; c Pathology Resident – PGY3, Hospital Universitário Getúlio Vargas, Manaus, Amazonas, Brazil; d Dermatologist, Residency Tutor, Tropical Medicine Foundation – Dr Heitor Vieira Dourado, Manaus, Amazonas, Brazil

**Keywords:** alcoholism, immunosuppression, mycology, sporotrichosis, tropical diseases

## Case report

**Fig 1:**
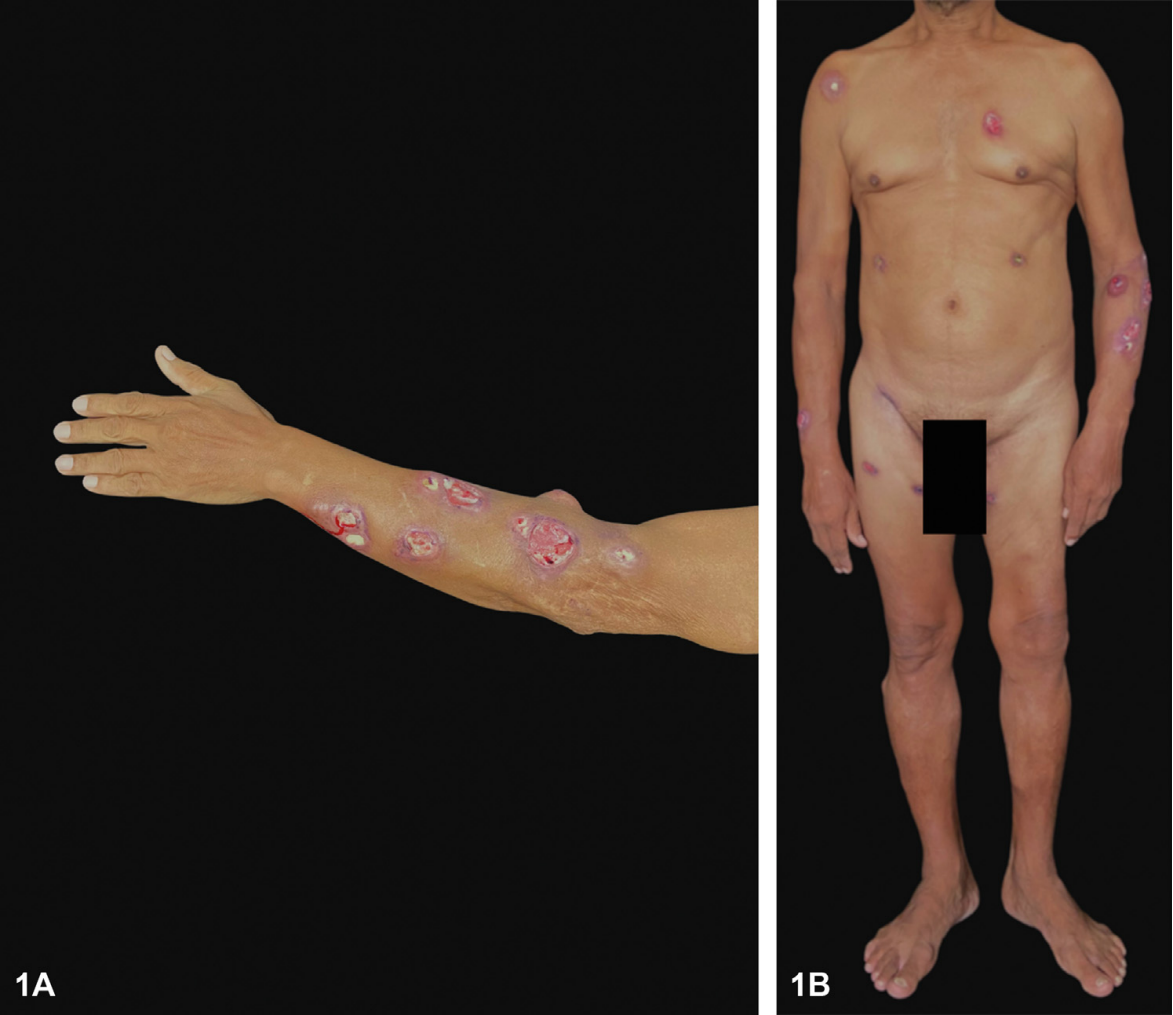

**Fig 2:**
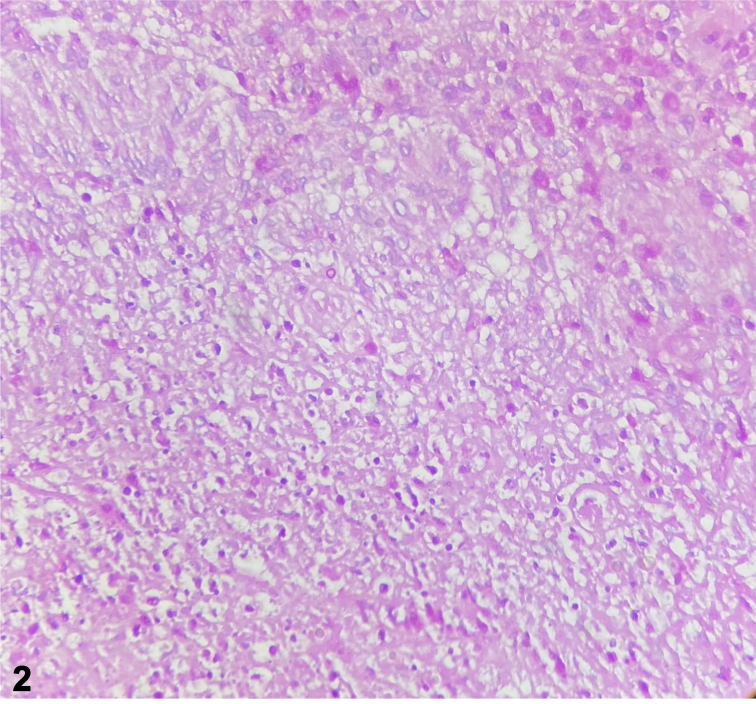

**Fig 3:**
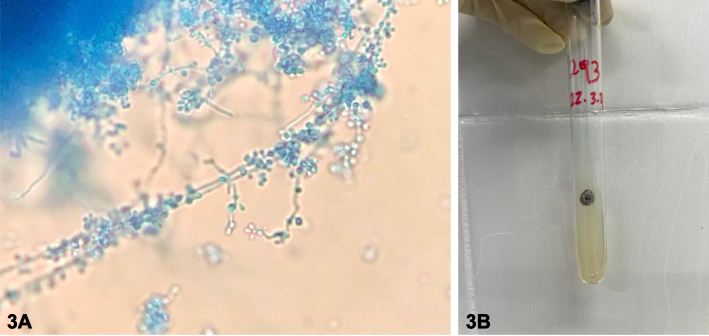

A 60-year-old male with history of alcoholic chronic liver disease and chronic hepatitis B virus infection presented with disseminated nodulo-ulcerative lesions for 2 months, without other symptoms. The patient was not taking any medication. Dermatological examination showed painful, erythematous nodules with central liquefaction and fistulization on the face, trunk, and limbs (Fig 1). In the histopathological exam, there was acanthosis, a diffuse mixed infiltrate in the dermis with multinucleated giant cells and yeasts-like bodies stained by periodic acid–Schiff (Fig 2). A blackish-brown filamentous colony was isolated in Sabouraud agar at 30 °C, with growth of septate hyphae with conidia arranged in a daisy shape (Fig 3).

**Question 1: What is the most probable diagnosis based on the medical history, clinical presentation, and histopathology?**

A. Cutaneous leishmaniasis
B. Nontuberculous cutaneous mycobacteriosis
C. Sporotrichosis
D. Histoplasmosis
E. Cutaneous lymphoma

**Answers:**

A. Cutaneous leishmaniasis – Incorrect. There was no marked plasmacyte infiltration and no morphological structures suspects of *Leishmania sp.* amastigotes were found.^1^
B. Nontuberculous cutaneous mycobacteriosis – Incorrect. There was no necrotic granuloma formation or acid-fast bacilli found in the histopathological analyses with the Wade-Fite technique. Also, there was no mycobacterial growth in culture samples at 25 °C and molecular testing for *Mycobacterium tuberculosis* was negative.^1^
C. Sporotrichosis – Correct. This is a case report of a disseminated sporotrichosis in a patient with alcoholic chronic liver disease and chronic hepatitis B virus infection, determining a subsequent immunosupression. Sporotrichosis is a chronic fungal disease caused by *Sporothrix sp.* (especially *Sporothrix brasiliensis* in Brazil) that grows in the soil and in the remains of plants, with infection usually secondary to local trauma. Cats are also considered a vector. Most disseminated forms of sporotrichosis are associated with immunosuppression and represent at least 5% of all cases. Disseminated nodules, papules, ulcers, or necrotic lesions are typical.^1^^,^^2^
D. Histoplasmosis – Incorrect. There were no morphological structures suspects of *Histoplasma capsulatum* yeasts using special histochemical stains. There was no growth of *H capsulatum* on the mycological culture.^1^
E. Cutaneous lymphoma – Incorrect. There were no atypical lymphoid cells in the histopathological examination.^3^

**Question 2: Which of the following microscopic characteristics is typical of *Sporothrix sp*. at 30 °C?**

A. Septate hyphae
B. Refractile branching fungal hyphae, like a rosary, on KOH 10%
C. Encapsulated yeast cells
D. Uniseriate or biseriate phialides
E. Piriform conidia arranged in a daisy-like fashion at the end of the conidiophore

**Answers:**

A. Septate hyphae – Incorrect. This is the microscopic appearance of filamentous fungi, such as *Aspergillus sp.* or *Penicillium sp.*, and is not typical of *Sporothrix sp.*^4^
B. Refractile branching fungal hyphae, like a rosary, on KOH 10% – Incorrect. Refractile branching fungal hyphae are usually observed in direct examination of causative agents of dermatophytosis.^4^
C. Encapsulated yeast cells – Incorrect. This feature is characteristic of encapsulated yeasts such as *Cryptococcus neoformans.* In the case of *Sporothrix sp.* at 30 °C, there are no yeast cells.^4^
D. Uniseriate or biseriate phialides – Incorrect. Their features can be found in *Aspergillus sp.* and *Penicillium sp.* Not being found in *Sporothrix sp.*^4^
E. Piriform conidia arranged in a daisy-like fashion at the end of the conidiophore – Correct. This feature is specific to *Sporothrix sp.* Under microscopy at 30 °C, the spores are found arranged in a daisy-chain arrangement at the end of the conidiophore.^4^

**Question 3: What is the most appropriate therapy to this case?**

A. Itraconazole
B. Amphotericin B
C. Potassium Iodate
D. Terbinafine
E. Meglumine antimoniate

**Answers:**

A. Itraconazole – Incorrect. Itraconazole is a fungistatic agent. It is considered the drug of choice in sporotrichosis and used in immunocompetent patients with the localized form as also in the immunosuppressed with systemic sporotrichosis without life-threatening disease.^5^
B. Amphotericin B – Correct. Amphotericin B, preferably in its liposomal form, is indicated in severe, life-threatening cases and especially in immunocompromised patients, until clinical improvement, when it should be replaced by itraconazole. It was the treatment of choice for this case. The patient had a good clinical response with scarring of cutaneous lesions and is currently in outpatient follow-up.^5^
C. Potassium Iodate – Incorrect. First choice for pediatric patients in localized mild forms. It can also be used in immunoreactive forms as it has antiinflammatory properties. In systemic or disseminated forms, it cannot be used in monotherapy.^5^
D. Terbinafine – Incorrect. First choice for mild to moderate cases in children older than 2 years. Useful in patients with comorbidities as it has fewer interactions with other drugs. It can be used in cases of absolute contraindication of itraconazole.^5^
E. Meglumine antimoniate – Incorrect. It is not a drug prescribed to treat sporotrichosis. Instead, it is useful in the treatment of leishmaniasis.^5^

## Conflicts of interest

None disclosed.
